# Making Research Accessible to the Developmental Language Disorder Community: A Mixed Methods Study Using the Nominal Group Technique

**DOI:** 10.1111/1460-6984.70096

**Published:** 2025-07-28

**Authors:** Emily Jackson, Janice Wijaya, Sanjana Bhatoolaul, Qi Xun Tan, Suze Leitão

**Affiliations:** ^1^ School of Allied Health Curtin University Perth Australia

**Keywords:** accessibility, developmental language disorder (DLD), dissemination, language disorders, plain language summary, research engagement

## Abstract

**Background:**

Accessing research can be difficult for individuals with developmental language disorder (DLD) and their supporting networks (e.g., family, speech–language therapists, and teachers). This challenge may be attributable to the DLD community's difficulty with searching and paying for scientific research and the complexity of language used in dissemination methods such as journal articles. It is important that members of the DLD community can access and understand research to facilitate the community's involvement in research and as a key part of building knowledge of DLD. To date, no studies have explored the DLD community's perspectives on their preferences for accessing and understanding research.

**Aims:**

This mixed methods study aimed to explore the DLD community's perspectives on how researchers can make their findings more accessible.

**Methods and Procedures:**

An international group of parents of children with DLD (including one parent who also had DLD) and a speech–language therapist (*n* = 9) participated in a nominal group technique process to share their perspectives. The group identified a range of methods that researchers could use to make DLD research more accessible to the community, which they discussed in depth and then ranked in order of preference. Consensus ranking analysis was used to identify preferred methods for research dissemination. The ranking exercise was supplemented by in‐depth discussions about research accessibility, which were analysed using qualitative content analysis.

**Outcomes and Results:**

Participants identified plain language summaries, flyers, infographics, and short videos as their preferred formats for making scientific research accessible to the DLD community. Qualitative analyses highlighted four main categories of recommendations for researchers, including the use of text‐based, visual (static), and multimedia (dynamic) approaches, as well as recommendations for making language adjustments and involving the DLD community in disseminating research findings.

**Conclusions and Implications:**

This study will help researchers better understand the DLD community's needs, enabling more effective dissemination of their findings to those most impacted by the research. In return, research findings are more likely to be translated into a form that the community can understand.

**WHAT THIS PAPER ADDS:**

*What is already known on this subject*

- Accessing research can be difficult for individuals with DLD and their supporting networks (e.g., family, speech–language therapists [SLTs], and teachers). No previous studies have explored the DLD community's perspective of their ability to access and understand the findings of research on the topic of DLD.

*What this study adds to the existing knowledge*

- This is the first known study to explore the perspectives of the DLD community on how researchers should disseminate findings in an accessible manner. The participants in our study ranked plain language summaries, flyers/pamphlets, infographics, and short videos as their preferred formats of disseminating scientific research.

*What are the clinical implications for this study?*

- We provide clear recommendations to researchers from the perspective of individuals from the DLD community. These include a range of formats and methods for increasing accessibility to, and engagement with, research findings. Improving accessibility may lead to empowerment of the DLD community and may aid their ongoing engagement in research processes.

## 1 Introduction

Developmental language disorder (DLD) is a lifelong neurodevelopmental disorder characterised by difficulties in the comprehension and/or use of language that cannot be attributed to any other biomedical conditions (e.g., autism spectrum disorder) (Bishop et al. 2017). These difficulties persist into adulthood and can impact functional communication across various contexts, such as academic and social settings (Botting 2020; Snowling et al. 2016). DLD has an estimated prevalence of 7% in English‐speaking countries (Norbury et al. 2016). Despite its prevalence, it remains under‐researched in comparison to other neurodevelopmental disorders (for every 100 individuals in the UK, an average of 0.13 papers related to DLD are published compared to 21.39 papers on autism spectrum disorder) (Bishop 2010; McGregor 2020). As well as there being limited research on DLD, there are barriers to the DLD community being able to access such research (St Clair et al. 2023). We use the term ‘DLD community’ to refer to individuals (both children and adults) with DLD, along with their families (e.g., caregivers, partners, siblings) and wider support networks, such as educators, healthcare professionals, advocates, and policymakers (Gasparini et al. 2025).

### 1.1 The DLD Community and Research Accessibility

It is important that the DLD community can access and understand research that has been conducted about them. This may support their sense of empowerment and ability to make evidence‐informed choices about DLD (Gasparini et al. 2025). However, the DLD community may find it difficult to access research due to the complexity of language used to describe scientific research findings, which are typically published in journal articles, books, and conference presentations and proceedings (Gasparini et al. 2025; Harold 2019). The jargon, complex sentences, embedded in‐text citations, and text structures of such publications may be especially challenging for individuals with DLD to understand due to their inherent challenges with comprehending vocabulary and sentence structures (Bishop et al. 2017; Snowling et al. 2016). Further compounding the difficulty with understanding research publications is that reading (e.g., decoding, reading fluency) can also be difficult for many people with DLD (see Adlof and Hogan for a summary) (Adlof and Hogan 2018), and that these challenges can affect both children and adults with DLD (Atchley et al. 2003). Overall, these potential difficulties with language and literacy may have a significant functional impact on the ability to read and process research for individuals experiencing them.

The language complexity of scientific publications may also impact accessibility for other members of the DLD community. This includes family members, who themselves may also experience language difficulties (Mountford et al. 2022), as well as ‘lay people’ without language difficulties who are unlikely to be familiar with much of the jargon used in publications (Edgell and Rosenberg 2022). Furthermore, time‐poor health professionals and educators may also find research reporting to be inaccessible, especially given the time involved in reading lengthy publications to stay abreast of the current research. To this end, at least 30% of research is considered to be not reported in a useful manner for stakeholders (Harold 2019; Chalmers and Glasziou 2009), with Bauer et al. (2015) highlighting that research publications often lack information on how to implement a new intervention or strategy, limiting the application of research into practice.

Another barrier to accessibility of research to the DLD community is the location of research publications. Most scientific research is published online in scholarly journals or presented at conferences, which are often inaccessible to the majority of the DLD community (St Clair et al. 2023). In particular, online research publications require a device with internet access, as well as the skills and knowledge to conduct an online search to retrieve relevant articles. If relevant articles *are* identified, very few articles from scholarly journals can be downloaded for free (‘open access’) (Harold 2019). With this in mind, some organisations are beginning to address research accessibility for the DLD community. One such organisation is the ‘*Engage with Developmental Language Disorder*’ (E‐DLD) Project, established in 2020 to connect academic researchers with people who have DLD (St Clair et al. 2023). E‐DLD has a growing database of people from the DLD community, who can freely access online talks, as well as plain language summaries of recent research. To date, however, no known study has explored the perspectives of people with DLD on their preferences for ‘consuming’ research, nor whether simplified research dissemination methods are effective in supporting their ability to access and understand research.

### 1.2 Plain Language Summaries

Plain language summaries are short, jargon‐free summaries of scientific publications designed to facilitate accessibility of research by a broad, ‘non‐expert’ audience (Edgell and Rosenberg 2022). These summaries can sometimes be found alongside peer‐reviewed journal publications; however, only 5% of biomedical and health journals have author guidelines on how to develop plain language summaries (Gainey et al. 2023). Plain language summaries play a vital role in facilitating research accessibility for people typically excluded from accessing and participating in research, such as those from marginalised groups (Rosenberg et al. 2023), and summaries may take varied forms, including written, graphical (e.g., infographics), or videos (Gasparini et al. 2025).

There have been some recent efforts to improve the use and effectiveness of plain language summaries for researchers. In 2022, Stoll and colleagues (2022) found that instructions for developing plain language summaries were varied, and that few studies explored the effectiveness of these summaries for improving accessibility, knowledge, understanding, and sense of empowerment among audiences. This emphasises the importance of developing guidelines tailored to the needs of different groups and contexts in collaboration with those groups, and of evaluating their effectiveness. One example of existing guidelines for creating plain language summaries is the Open Accessible Summaries in Language Studies (OASIS) initiative (Marsden et al. 2018). OASIS offers a structured approach for researchers to produce concise, one‐page summaries of their work on language learning, use, and education. These one‐page summaries are then hosted in a publicly accessible database. There exist guidelines for creating other dissemination outputs, such as visual (Ibrahim 2018) and video (King et al. 2023) abstracts, which focus on making research communication more effective for both academic audiences and the public. However, existing guidelines do not specifically address the needs of individuals with communication disorders.

Hinckley and El‐Khouri (2023) compiled guidelines for publishing ‘aphasia‐friendly’ research, which included easy‐read summaries, graphical abstracts, and video abstracts no more than 5 min long. Recently, Gasparini and colleagues (2025) published the first known guidelines to support dissemination of accessible research to the DLD community. These were developed following the OASIS guidelines and involved consultation with one person with DLD, an implementation scientist, and a speech–language therapist (SLT). However, in‐depth perspectives of the DLD community were not explored. Therefore, in the current study, we sought to explore the DLD community's perspectives on methods for research dissemination, with a focus on gathering ideas on how research can be made more accessible to them.

Exploring the DLD community's perspectives on increasing research accessibility is driven by the patient and public involvement (PPI) model (Arumugam et al. 2023). PPI involves inviting individuals from the target population to be an active part of the research process, rather than being ‘passive’ participants. Ensuring that individuals with DLD can access and understand research has the potential to build knowledge of their condition and may also empower them to contribute to future research efforts (Gasparini et al. 2025). We suggest that if the DLD community can be actively engaged in the research process, their insights may help shape more relevant, accessible, and impactful findings, leading to improved awareness, treatment, and long‐term support for DLD (St Clair et al. 2023). By framing the research within a PPI model, we aimed to increase the relevance, accessibility, and efficiency of dissemination to the DLD community (Arumugam et al. 2023; Hersh et al. 2015).

## 2 Method

### 2.1 Research Design

In this mixed methods study, an online nominal group technique (NGT) was adopted to gather quantitative and qualitative data in a group setting. As described in further detail below, participants generated and then ranked preferred ideas for how researchers could disseminate research findings (quantitative). Additionally, participants took part in in‐depth discussions on the topic of research accessibility methods (qualitative).

### 2.2 Participants

We sought participation from individuals from the DLD community, which included (i) adults who had been formally diagnosed with DLD, (ii) family members or other personal support persons (e.g., parent/caregiver, partner, close friend) of an individual with DLD, or (iii) professionals (e.g., teachers, SLTs) who work with children or adults with DLD. We feel that the challenges faced by the DLD community, in terms of their language skills and difficulties accessing scientific research (e.g., paywalls), are not specific to one geographic region. For this reason, and because this was the first study to explore DLD perspectives on the topic, we chose to recruit internationally. To take part, participants needed to be able to speak English fluently and identify as having DLD (whether or not they had a formal diagnosis) or be a member of an individual with DLD's personal network (e.g., parent/caregiver, sibling, friend, partner). Additionally, professionals (including teachers or health professionals) were able to take part if they provided regular support for individuals with DLD (e.g., in a classroom or clinical setting).

Participants were sampled through purposive and snowball sampling. The study was advertised through an easy‐read flyer, with an embedded QR code to an online expression of interest form, which was posted to the researchers’ social media accounts and via DLD networks (e.g., E‐DLD). This resulted in a total of nine participants: one SLT and eight parents of children diagnosed with DLD (one father and seven mothers), one of whom also described themselves as having DLD. Eight of the participants (seven parents and the SLT) were in Australia (spanning four states or territories), and the ninth participant was in Canada.

### 2.3 Procedure

This study was approved by the Curtin University Human Research Ethics Committee. Participants expressed interest in the study through the completion of an online survey on the Qualtrics platform (Qualtrics 2024). One researcher contacted participants to confirm their eligibility, answer questions about the study, obtain written consent, and gather information on whether they had any accessibility needs to support their participation. Participants provided consent for the sessions to be video‐ and audio‐recorded and transcribed to support qualitative analysis of group discussions.

**FIGURE 1:**
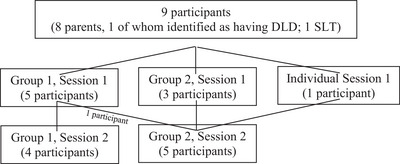
Overview of participants in each NGT group. NGT, nominal group technique.

Given the differing availabilities of the participants, the participants were divided into two groups (see Figure 1), who each took part in two 1‐h sessions held 2 weeks apart. One participant from Group One could not attend their group's second session due to family commitments; therefore, they joined Group Two's second session. Another participant could not join a group for the first session. As such, they completed an individual interview and then joined Group Two's second session. One participant from each group did not complete the ranking exercise (due to reasons such as needing to leave the session early due to work commitments). All sessions were held online via Microsoft Teams and were video recorded and transcribed using the built‐in software.

### 2.4 NGT

NGT is a structured variation of a focus group that guides discussion around a single topic or central research question (Dunham 2006). The research question guiding the NGT methodology in this study was: ‘*What are the best ways to make scientific research accessible for the DLD community?*’ NGT consists of four stages: (1) generating ideas, (2) recording ideas, (3) discussion of ideas, and (4) voting on ideas (ranking preferences).

Within the field of speech–language therapy, NGT has been used with other populations experiencing communication challenges, such as stroke survivors (and relevant health professionals), for purposes such as identifying the core components of rehabilitation in the home (Fisher et al. 2021). The NGT method was chosen because it provides a structured yet inclusive approach to gathering diverse insights into a central research question. NGT facilitates equitable contribution among participants by allowing each member to contribute ideas independently prior to taking part in group discussion. Additionally, the idea ranking process helps to identify the most impactful strategies as a group, which is important for identifying community‐driven methods for enhancing research accessibility (Dunham 2006). Due to the interstate and international location of participants, the NGT sessions were conducted online.

To comprehensively explore the research question and allow participants time to consider their ideas, we conducted NGT over two sessions (Fisher et al. 2021). That is, the two groups each participated in two 1‐h sessions. The topic and structure of the sessions were consistent for both groups. Each session was led by one facilitator. Four other members of the research team were present in the sessions, with the primary purpose being that individualised support could be provided to each participant during the sessions as needed. For instance, if participants required help to understand the activity, express ideas, or share concerns, then they could enter a ‘breakout room’ with a researcher, separate from the main group. The additional researchers also provided support with the technical requirements of running online sessions (e.g., recording sessions and taking notes).

**Session 1**. The first online session focused on Stages 1 and 2 of NGT. After informal introductions to each other within the group, the facilitator shared PowerPoint slides that included plain English information about the aims of the session and were used to guide the discussion. First (Stage 1), the facilitator facilitated a discussion on the group rules, study overview, and research question. Then, the participants were given time to silently and independently reflect on the question and generate their own ideas. During this time, the facilitators supported participants by clarifying any thoughts about the research question. If a participant requested individualised help away from the group (e.g., if they were unsure of the task requirements or needed support to formulate ideas), a facilitator would move to a ‘breakout room’ in Microsoft Teams.

Next (Stage 2), participants were invited to share ideas they had generated with the group using a ‘round robin’ style (whereby there were no interruptions or debates on each other's ideas) (Fisher et al. 2021). Prompts were provided by the facilitator to encourage more detailed responses where needed. Following the first session, the research team compiled the ideas generated by the group using plain English, to be discussed further in session 2. Participants were invited to email the research team with any ideas or queries between the first and second sessions.

**Session 2**. In this session, Stages 3 and 4 were conducted. First (Stage 3), the facilitators re‐stated the aims and group rules before presenting the participants’ summarised ideas from session one. This step aimed to facilitate credibility and ensure ideas from Session 1 had been accurately interpreted by the researchers. The facilitator then invited group discussion in which participants had the opportunity to add, remove, or clarify the ideas before ranking took place (Dunham 2006). The list of ideas was updated accordingly and imported into an online survey in the Qualtrics platform (Qualtrics 2024).

Subsequently (Stage 4), the participants were given the link to the survey and spent 5–10 min individually ranking the ideas from most to least preferred methods that researchers could use to disseminate their research findings in an accessible way to the DLD community. The session ended with researchers re‐stating the aims of the study and describing how the results would be analysed and disseminated. The participants were asked to share any final thoughts, ask questions, and give feedback on the sessions.

### 2.5 Analysis  

The quantitative data for each of the two groups were analysed separately. That is, Group One and Group Two ranked ideas that they generated within their own group. Each participant individually ranked the ideas generated within their group from most to least preferred. Consensus ranking analysis was then used by summing the rankings (ordinal scores) across participants within each group.

The group discussions from the first and second NGT sessions for each group were recorded and transcribed verbatim and then analysed using inductive qualitative content analysis (Elo and Kyngäs 2008). An inductive approach is most appropriate in the context of generating new data, as opposed to a deductive approach, which analyses and compares data to previous research. First, three authors immersed themselves in the data by reading all written responses several times. The authors then made notes throughout the transcribed responses to start making sense of the data (Elo and Kyngäs 2008).

Subsequently, open coding of transcripts from Groups One and Two was completed by three authors to generate codes that described the key concepts, ideas, or patterns. Given the similarity in codes across the two groups, all transcripts (sessions one and two from both groups) were coded together. Subsequently, the codes were discussed by all five authors, and labels for codes were amended for clarity where needed. Then, the codes were collaboratively grouped into categories and subcategories, where four of the 57 codes were disputed but resolved via discussion among the team. This resulted in a total of 21 subcategories, 18 generic categories, and four higher‐order categories.

## 3 Results

### 3.1 Ranking of Ideas (Quantitative Data)

**FIGURE 2:**
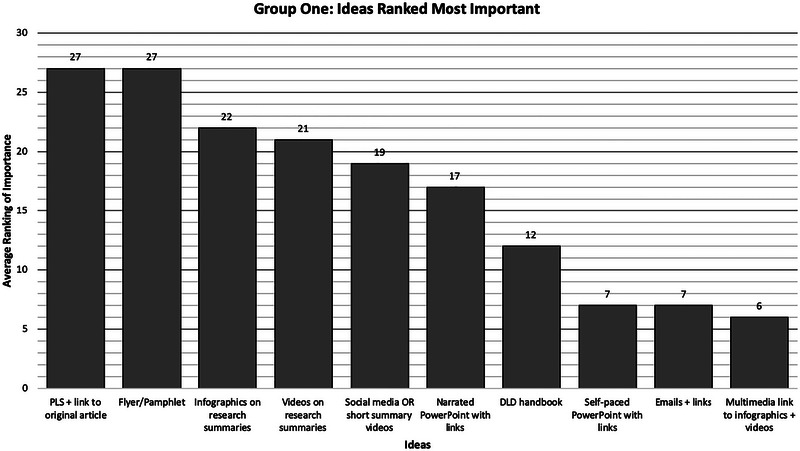
Group One ideas ranked as most important in the second NGT session, with numeric values denoting the summed preference score aggregated across participants. NGT, nominal group technique.

Three out of four participants in Group One (75%) and four out of five participants in Group Two (80%) participated in the ranking activity during the second NGT session, where ideas were ranked by level of importance or preference. Group One generated 10 ideas. The three participants ranked the 10 ideas from most to least preferred (most preferred = 10, least preferred = 1). These rankings were then summed across participants. Given that there were three participants and 10 ideas, the highest possible summed ranking for a score would be 30 (which would indicate that the idea was rated 10—*most preferred*—by all three participants). The results are presented in Figure 2 (the numeric value at the top of each bar indicates the summed score for that idea).

As shown in Figure 2, Group One identified two ideas as being equally preferred: (1) Research summaries should be provided as a plain language summary with a link to the original article, and (2) research summaries should be presented as flyers or pamphlets. The idea ranked as least preferable was a multimedia link that guides viewers to further formats, including infographics and videos of research summaries.

**FIGURE 3:**
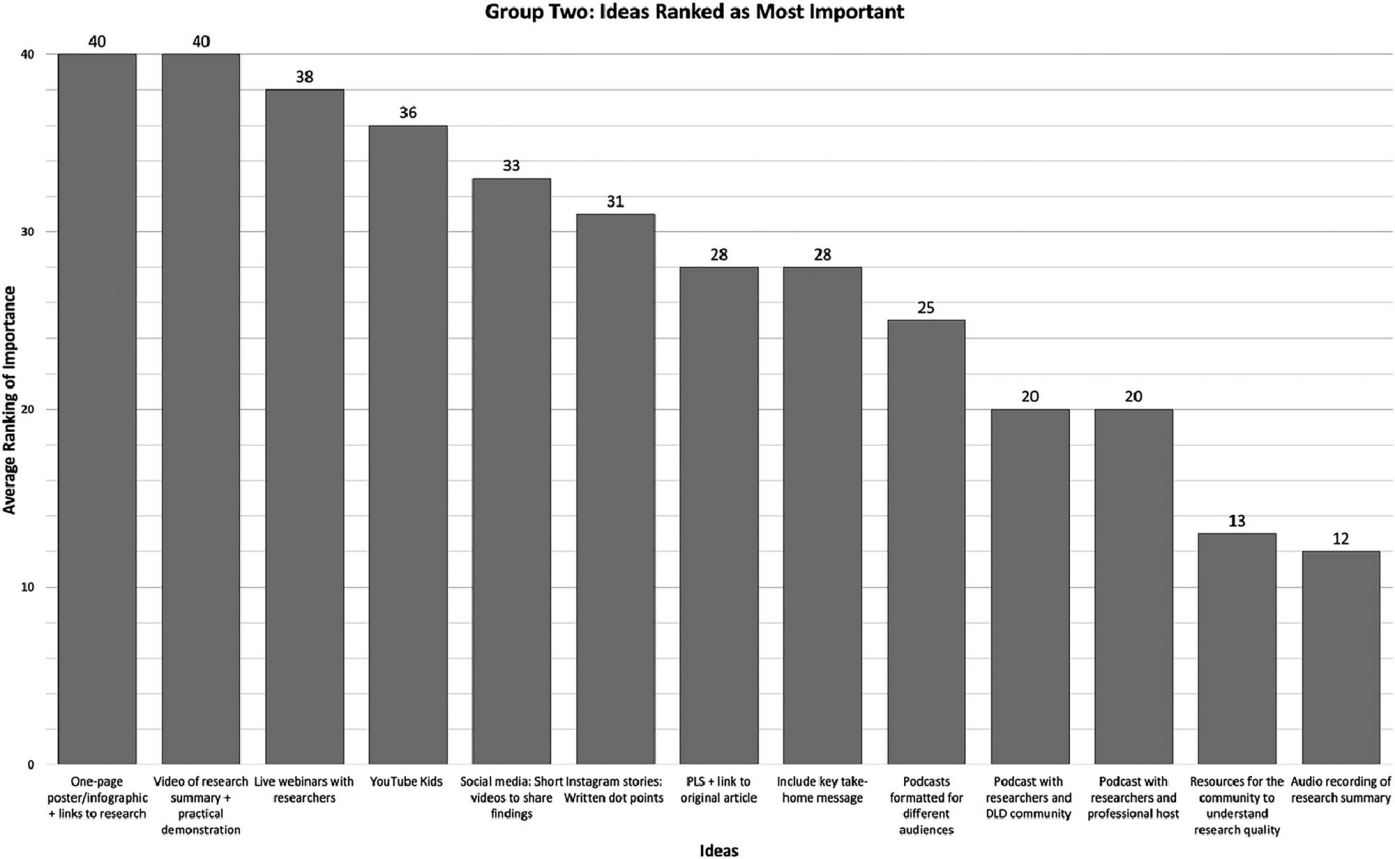
Group Two ideas ranked as most important in the second NGT session, with numeric values denoting the summed preference score aggregated across participants. NGT, nominal group technique.

Group Two generated 13 ideas (see Figure 3). The four participants ranked the 13 ideas from most to least preferred (most preferred = 13, least preferred = 1), with rankings summed across participants. The highest possible summed ranking score was 52.

Like Group One, the second group equally ranked two ideas as being most preferred: (1) *Research should be disseminated via a single page poster or infographic with a link to the original article, and (2) research should be disseminated via a video summary that includes a practical demonstration of the topic* (e.g., a demonstration of an intervention approach that could be used for people with DLD). Participants chose audio recordings of research summaries as the least preferred idea.

### 3.2 Discussions About Research Accessibility Preferences (Qualitative Data)

**FIGURE 4:**
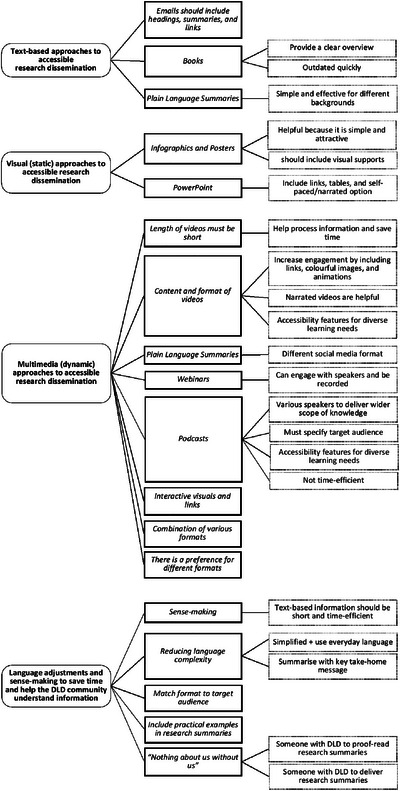
Perspectives from the DLD community participants on how to make research more accessible, as grouped into higher‐order categories (left), generic categories (middle), and sub‐categories (right). DLD, developmental language disorder.

The findings from the inductive content analysis are presented descriptively below. Following the process of open coding, in which 52 codes across the two groups were generated, the codes were categorised into subcategories, then into generic categories, thereby generating the final higher order categories (see Figure 4).

Based on participant discussions throughout the NGT sessions, we identified four higher order categories relating to the possible methods for accessible research dissemination for the DLD community: (1) *Text‐based approaches*, (2) *Visual (static) approaches*, (3) *Multimedia (dynamic) approaches*, and (4) *Language adjustments to save time and help DLD community understand information*. These categories are presented with illustrative quotes in the sections below.

### 3.3 Text‐Based Approaches to Accessible Research Dissemination

The first higher‐order category related to researchers presenting their findings via text‐based dissemination methods, such as emails, books, and plain language summaries.

#### 3.3.1 Emails

Across the groups, a common suggestion was that researchers could disseminate their findings via email. The participants explained that emails should include clear headings and summaries:

*‘In the e‐mail itself, you'll get a heading of what the paper was, a brief synopsis of what the paper covers, and then it takes you to a page that gives you a breakdown… of the full paper, and then references back to the actual full paper if you need it. Just that way I can actually read the papers that are relevant to [my child].’*

Additionally, it was suggested that emails should contain a link for readers to access the full version of the article: *‘For any papers that they've published, they send out links to that paper.’*

#### 3.3.2 Books

There were some suggestions that researchers could present their findings as a book. Some of the benefits that were mentioned were that books may provide a clear summary of the topic, and books can be useful for community members where internet access is limited, such as rural areas. This was exemplified by the quote:

*‘I remember reading a book for sensory processing disorder back when [my child] was about three and it was so good to just read, you know, 300 pages… just really have a big read of this book to really understand what was going on for her.’*

In contrast, some participants reasoned that books *‘can get outdated quickly,’* and that they are costly because publishers *‘have to make some money back.’*

#### 3.3.3 Plain Language Summaries

The participants highlighted that plain language summaries could help readers understand the main findings of a study and then access the link to the original article for more details. For example: *‘I think if you can get a snapshot of something there with the link for people like me who might like to read the original article and the details.’* There was also the idea that *‘plain language summaries would get across range of people from different educational backgrounds,’* as they are simple and quick to read.

### 3.4 Visual (Static) Approaches to Accessible Research Dissemination

This higher‐order category pertains to researchers using a combination of text and static visual information through formats such as infographics, posters, and slideshows (e.g., PowerPoint).

#### 3.4.1 Infographics and Posters

In this sub‐category, participants mentioned the use of infographics, brochures, pamphlets, and factsheets. Participants reported that these formats should be made attractive by including pictures and images that support the understanding of written information. This will *‘stick out to them [readers]’* and motivate ‘*them [readers] to read it all’*. Participants also highlighted that written information in these formats should be kept short and concise.

#### 3.4.2 Slideshows

Slideshows (using software such as PowerPoint) were highlighted as a potentially useful visual format to present research information and could be designed in a way that readers can work through in a self‐paced manner. Specifically, it was suggested that slideshows should include a table of contents for readers to navigate and *‘skip through to get the information they need.’* Additionally, participants highlighted that complex words and concepts presented on the slides should be explained through a link that directs them to a definition and more information to support in‐depth understanding. In contrast to ‘self‐paced’ slideshows, participants suggested that researchers could present their findings via researcher‐narrated slideshows, by recording themselves talking through their findings on each slide. Participants expressed interest in both self‐paced and researcher‐narrated methods if the slides were compact and short. One participant preferred narrated slideshows, as noted:

*‘I think for people with DLD that's a great one because it kind of gives you the immersive reader option. At the same time, someone explaining it in a slightly different language, and then you can read it as well.’*

### 3.5 Multimedia (Dynamic) Approaches to Accessible Research Dissemination

Within this category, we identified a combination of ideas about researchers disseminating their findings using elements of audio, visual, and text formats. Participants made several recommendations regarding the length, content, and format of videos, use of interactive visuals and links, social media, and webinars. The idea of having personal preferences for engaging with differing multimedia formats was also raised.

#### 3.5.1 Length of Videos

A key idea identified was that videos presenting on research findings should be *‘short and very simple,’* and that there is the tendency to not *‘listen anywhere past three minutes.’*

#### 3.5.2 Content and Format of Videos

Participants noted that interactive links should be added to increase viewers’ engagement. One suggestion was to include a link encouraging viewers to consider the relevance of the information to their own experiences, as well as a link to the original article:

*‘The way I think best catches people is simplistic videos… “have you experienced this?” and a connection to a click button to “this is what this means,” and it might connect through the research for people like me who might like to read it.’*

There was also the recommendation to use animations and cartoons, which might support children with DLD to be more engaged in learning about DLD. Participants highlighted that videos narrated by researchers could be particularly useful in building a sense of confidence and trust in the information being presented:

*‘A video recording of them speaking and you could see the visual, you see everything. I prefer to see the physical person who is speaking, because sometimes people can speak through audio recording, but you don't know if it's legit or not.’*

Alternately, there was the suggestion about the potential power of having a public figure or celebrity narrate or host the videos or audio recordings. Participants discussed that this could help to increase viewers’ attention and may also contribute to improved awareness of DLD:

*‘Whenever you see video clips that's narrated by let's say a footy player or an actor, anyone that people know with a nice voice, they might have no idea about the subject, but it doesn't really matter. It seems to draw a lot of attention to that topic.’*

Additionally, it was suggested that videos should include accessibility features to support people with diverse communication needs. For instance, one participant referred to video resources on DLD they had seen recently, stating that *‘the videos had sign language on the side. That was incredible.’*

#### 3.5.3 Social Media

Responses within this category included suggestions to deliver research summaries through different social media platforms. This included using a single social media post format with dot points (‘*just one point from the research that you can use in the classroom, and then a link to the article’*), Instagram reels (‘*reels just as a way of introducing people’*), posting on [Instagram and Facebook] stories (‘*stories where you show your research still in text, but either 10 stories with links’)*, and Facebook posts (‘*longer Facebook posts’*).

#### 3.5.4 Webinars

Participants suggested that webinars are a helpful way to disseminate research information, as they tend to encourage interaction between the researcher and audience. As such, webinars were described as an opportunity to ask questions and be actively involved in research:

*‘I like the idea of webinars because there's always an opportunity to be able to ask questions, whether that's putting it onto a chat if you don't want to speak up, or to be able to speak up if you want to.’*

Webinars were also described as potentially useful because members of the DLD community can watch them in their own time and pace: *‘I think it would be smart to do something that's live that's recorded and then people can then choose to watch if you missed it.’*

#### 3.5.5 Podcasts

It was suggested that researchers could have their own podcast, or be guests on well‐known podcasts, in which they share information about themselves and discuss key findings from their research studies: *‘The researchers are doing the podcasts and give a little information about their research project.’* It was also suggested that podcasts should feature more representatives from the DLD community:

*‘If it's a discussion on a parent with a child with DLD, or a student with DLD talking, that's great to get that perspective. And even teachers or pediatricians, talking about the research, because what we really need is, you know, what's going to work best so we know how to support students with DLD in the classroom.’*

Additionally, participants described how it would be useful for podcasts to be labelled and categorised based on the target audience (e.g., health professionals, teachers, and parents) so that listeners can easily find content most relevant to them. Furthermore, accessibility features in podcasts were recommended to support the diverse needs of the DLD community (e.g., a written transcript of podcasts). While podcasts were mentioned to be potentially beneficial for people with visual impairments, they were critiqued for their length, and that some members of the community ‘*don't have the patience*’ to listen through the whole podcast. Relatedly, the idea was raised that podcasts reinforce a ‘*one dimensional view’* of information, and that other interactive formats (e.g., webinars and workshops) may be preferable, as the audience can actively ask questions of the researcher.

#### 3.5.6 Interactive Visuals and Links

It was recommended that digital visual materials (e.g., infographics) should have interactive elements, including embedded icons with links that provide further details, including information about the source(s) of the material. This was suggested as being important in supporting comprehension of the information and increasing trustworthiness:

*‘Adding the link onto the graphic, where they can click on that little picture and then it'll take them to that research information. So, they don't have to be saying “oh, is this legit?” Just click on the graphic and then it goes back, and forwards and it can go through a storyline.’*

#### 3.5.7 Combination of Various Formats

An idea raised in the groups was the recommendation that a combination of formats for summarising research *‘works well for Neurospicy people,’* as this provides them with multiple ways to process the information. A specific example was that research could be presented as an infographic with a corresponding short video clip of a person *‘explaining things in a language that you can understand.’*

#### 3.5.8 Preference for Certain Formats

Relatedly, discussions revealed that individuals may have strong preferences for certain formats because they align with their communication strengths and needs. For example: *‘if you're doing video clips, you wouldn't need infographics. They'd [videos] be more interesting from my perspective.’* A different perspective offered was that books should not be used to disseminate research as they are too ‘tedious’ compared to formats like infographics or pamphlets, although it was acknowledged that perhaps *‘that's just me being way too lazy to read a book.’*

### 3.6 Language Adjustment to Save Time and Help DLD Community Understand Information

While all previous categories relate to the types of formats that may be used to present information, this category relates to the language used to present research, regardless of the format (e.g., videos, infographics, written summaries). Specifically, the idea underpinning this category was the researchers should focus on ‘sense‐making,’ reducing language complexity, matching format to the target audience, and including practical examples. Furthermore, a key recommendation was that researchers should work alongside and continually learn from the DLD community in their effort to increase accessibility of research.

#### 3.6.1 Sense‐Making

The group discussed the notion that researchers have a responsibility to convey complex findings from a study in a way that makes sense to the audience. To this end, a key suggestion was that research summaries should be kept short and concise (‘*chunks of information,’* and *‘as a person who's got DLD, I need things that are short*’), and that full‐length papers should always be accompanied by an easy‐read summary of key ideas (i.e., ‘*a brief synopsis of what the paper covers*’). Participants specified that researchers could aim to make their written summaries approximately one paragraph long to make them time‐efficient to read (*‘instead of 12 pages and 20 references, you have a bit more than a paragraph’*).

#### 3.6.2 Reducing Language Complexity

Another idea generated among the groups was that information in research summaries should be simplified and easy‐to‐understand using plain language that could be understood by non‐researchers. This was because the language used in most research articles is too complex. For example: ‘*the issue with academic writing and research article is the language is not for everyone, which is also barrier for families who have a language disorder’* and that publications are ‘*hard to understand for some people, because they use vocabulary that can be advanced, especially for a parent that's not used to reading science articles.’*

Therefore, it was suggested to ‘use simpler vocabulary to help parents of children with DLD or even people with DLD who want to inform themselves on their own condition.’ This was echoed by the statement, ‘as someone who's got DLD, I need things that are very simple.’ It was also noted that plain language summaries help relay information back to children with DLD: ‘Plain language is great because either way I'm going to explain it to [my child].’ Moreover, participants emphasised that a key take home message should be included at the end of article to provide a summary of the research summary.

#### 3.6.3 Match Format to Audience

The participants highlighted the need for researchers to identify the target audience and adjust the formats to meet their needs. This was described as necessary to increase the opportunity to reach more readers (*‘just misses so many of the cohort or people who would benefit’*). This was raised as important when disseminating research to younger people with DLD:

*‘It's one thing for adults, but if you want to share a child or a teenager, for them to understand, you need to find another way to give out the information. Because sometimes kids don't want to look at graphics, sometimes they like to listen.’*

#### 3.6.4 Include Practical Examples in Research Summaries

Participants also recommended that researchers should include practical or ‘*tangible’* examples in research summaries: ‘*like presentations at a conference but showing this is me doing the intervention and this is what works.’* This was regarded to be particularly beneficial for parents and teachers to build their understanding of how to apply the findings at home or in their professional practice.

#### 3.6.5 Nothing About Us Without Us

‘Nothing About Us Without Us’ refers to the fundamental principle of representation (Koontz et al. 2022). It emphasises that policies, decisions, and discussions about a particular community should not be made without their active involvement and input. Participants in the groups highlighted that it is crucial to have people with DLD involved in the development of research summaries, including the processes of designing formats through proofreading the final product, before disseminating anything to the community. This idea was embodied by the quote: ‘*I would really like to see somebody with DLD be involved in that process.’* Involvement of people with lived experience of DLD was deemed necessary for research to be effectively translated to those without DLD within the community (*‘people like me* [without DLD] *don't know what it's like to be in their shoes, so if they could help?’*). Similarly, there was the idea that someone with DLD could be involved in developing (e.g., writing and/or presenting) research summaries. This aligned with the notion that research findings would resonate with the DLD community if they were shared through the lens of those with lived experience:

*‘I think people with DLD talking about their experiences on those shows so that people can resonate. I guess a parent who is willing to have their child on there to then talk about DLD and those types of things.’*

## 4 Discussion

The purpose of this study was to explore the perspectives of people from the DLD community on how researchers could make research more accessible. In this study, eight parents of children with DLD (one of whom also had DLD) and one SLT took part in a NGT process, where they generated recommendations for researchers and ranked ideas on how to improve research accessibility. There was a broad range of ideas on how researchers could format or present their research findings in ways that are easy to read, view, and/or listen to and understand by individuals with DLD, their families, and broader support networks (e.g., educators and health professionals).

We found that the highest‐ranked formats for disseminating scientific research to the community included plain language summaries, pamphlets, infographics, and short videos of research summaries. Perhaps unsurprisingly, these are all simplified formats that incorporate simple language, which would make them easier and quicker to engage with compared to ‘traditional’ scientific dissemination formats like journal articles. Furthermore, most of the preferred formats included visual supports (e.g., icons), unlike the lowest‐ranked format of audio recordings, which are likely to pose challenges for those with oral language comprehension difficulties (Botting 2020; Snowling et al. 2016). Similar findings were identified by Hinckley and El‐Khouri (2023), who conducted a systematic literature search to underpin guidelines for aphasia‐friendly dissemination methods. Hinckley and El‐Khouri also recommended formats such as plain language summaries and graphical summaries, and that they should be short, simple, and clear, echoing our findings from the DLD community.

Our findings build on preliminary work conducted by Gasparini et al. (2025), who developed a set of guidelines on how to write plain language summaries for the DLD community with input from one individual with DLD, one SLT, and an implementation scientist. They made specific recommendations around the preparation of written and video summaries. The recommendations made by our participants aligned with many of the guidelines suggested by Gasparini and colleagues, such as the length of written summaries to be a maximum of two pages and include smaller chunks of information. Similarly, Gasparini and colleagues recommended that videos be short, which was echoed by our participants (who specifically recommended videos be a maximum of 3 min). This recommendation is likely to be especially important for those who have DLD, given that many may experience reduced working memory capacity and sentence comprehension compared to individuals without DLD (Montgomery et al. 2021). These factors likely contributed to our participants preferring dissemination methods with short formats (e.g., short videos and social media posts) that place less demand on working memory and cognitive processing. These shorter videos are also likely to facilitate accessibility for other members of the community without language difficulties, such as time‐poor families and professionals (Gasparini et al. 2025; Harold 2019). An important point of difference between our work and that conducted by Gasparini and colleagues was that we engaged a broader range of members from the DLD community. We also sought to generate ideas on dissemination methods rather than seeking feedback on those that had previously been created. Additional recommendations for research accessibility identified in our study not covered in Gasparini and colleagues’ guidelines included ideas such as books, embedding practical examples, inclusion of individuals with lived experience and/or public figures in presenting summaries, and webinars.

Regardless of the format, our participants recommended that researchers simplify the language complexity within research summaries, such as using simpler vocabulary and less‐complex sentences. This was important to facilitate sense‐making among individuals with DLD but was also seen as important to ‘non‐experts’ who may also have difficulty understanding technical jargon (Edgell and Rosenberg 2022). This is in line with previous studies that proposed removing unnecessary ‘distractors’ and utilising high‐frequency words and simple sentences (Hinckley and El‐Khouri 2023; Rose and Meyer 2002), coupled with visual aids, to support comprehension (Edgell and Rosenberg 2022; Hinckley and El‐Khouri 2023). While these recommendations were often discussed as being ways to support individuals with DLD, it is likely that other stakeholders (e.g., family members, educators) may benefit. We should not assume that members of the broader DLD community have the skills to interpret a typical research article, as some may have reduced health literacy, for instance, which may contribute to difficulties reading at an academic level (Hersh et al. 2015). By considering the accessibility needs of the DLD community, researchers may contribute to efforts to challenge ableism, noting that traditional methods of sharing research inherently exclude the DLD community. Making research findings more inclusive to the community shifts the focus from expecting individuals to adapt to inaccessible systems, to changing those systems to be more equitable (Lundberg and Chen 2024).

Our findings also highlight the importance of increasing engagement in research findings in the broad community so that they feel empowered in their understanding of the latest evidence. This is an important part of evidence‐based practice so that individuals engaging with services (e.g., speech–language therapy) can seek to understand the evidence behind the approaches used in assessment and intervention and can ask informed questions (Greenhalgh et al. 2023). Furthermore, increasing the community's engagement with research is seen as an important component of PPI, in which stakeholders are involved meaningfully in research (e.g., identifying research priorities, developing and reviewing research proposals, and designing study materials) (Arumugam et al. 2023). As discussed by Gasparini et al. (2025), ‘while stakeholders should not need to be research consumers to effectively take part in co‐design activities, their participation in co‐design activities may be supported if they have some understanding of the existing research, to situate new information with their lived experience and needs’ (3). As such, improving research accessibility may be a key part of enhancing PPI and underpinning more equitable collaboration between researchers and community members, which may in turn contribute to research being conducted in ways that reflect the needs of consumers (Rosenberg et al. 2023).

As well as describing ways to support research accessibility for the DLD community as a whole, our participants highlighted their individual preferences, with some debate on the ‘best’ methods. Ranking data reflected this variation, with videos being ranked first in Group Two but fourth in Group One, and plain language summaries ranked first in Group One but seventh in Group Two. These differences may relate to individual communication strengths (e.g., stronger reading skills in Group One), though exploring ranking rationale was beyond the scope of the NGT process. This finding aligns with the heterogeneous nature of DLD (Bishop et al. 2017) and general diversity in how individuals prefer to engage with content. A key message derived from these results is that there is no ‘one size fits all,’ but rather a range of formats and modalities should be offered, and that researchers can follow some core principles for enhancing accessibility.

### 4.1 Limitations and Future Directions

Our use of a purposive and snowball sampling strategy may have led to participation bias in that participants already knowledgeable on the topic may have volunteered to participate (Palinkas et al. 2015). Additionally, most of our participants were parents of children with DLD located in Australia, which likely resulted because of our sampling strategy involving advertising within our extended professional networks. All participants also had English as their primary language, and we did not collect data on whether participants spoke different languages, reducing the generalisability of our findings. Furthermore, we did not receive any expressions of interest from young people with DLD to participate and had limited time in which to recruit and collect data. Future research should seek perspectives from participants of various ages because children also have a right to seek understanding of research that concerns them. Additionally, broader recruitment from a wider range of countries with individuals from different language and cultural backgrounds would be important to guide improved research accessibility to diverse communities.

## 5 Conclusions

It is important that researchers convey complex findings from a study in a way that makes sense to the readers (e.g., the DLD community) who are most impacted by the research. To our knowledge, this is the first study to explore the perspectives of the DLD community on how researchers should make their findings more accessible. In summary, there were key recommendations about the use of certain formats for presenting research to the community, including text‐based methods (e.g., plain language summaries and email summaries), visual (static) methods such as infographics, and dynamic multimedia formats, such as narrated slideshows, social media video clips, and podcasts with supplemental materials. Regardless of the format used, there were foundational principles underpinning the presentation of research findings to aid accessibility, such as simplifying vocabulary, using shorter and less complex sentences, and providing opportunities for audiences to easily locate more information. Importantly, to accommodate individual preferences and communication strengths, researchers should disseminate findings using a variety of formats. Furthermore, our participants called for increased involvement of members of the DLD community in the research dissemination process, including designing dissemination materials and presenting the content, to increase relevance, meaningful engagement, and empowerment for the community. The findings of this study may provide researchers with a greater understanding of how to effectively disseminate their findings to key stakeholders who are arguably most impacted by the findings of such research.

## Conflicts of Interest

The authors declare no conflicts of interest.

## Data Availability

Data may be made available by the authors upon request.

## References

[jlcd70096-bib-0001] Adlof, S. M. , and T. P. Hogan . 2018. “Understanding Dyslexia in the Context of Developmental Language Disorders.” Language, Speech & Hearing Services in Schools 49, no. 4: 762–773. 10.1044/2018_LSHSS-DYSLC-18-0049. 30458538 PMC6430503

[jlcd70096-bib-0002] Arumugam, A. , L. R. Phillips , A. Moore , et al. 2023. “Patient and Public Involvement in Research: A Review of Practical Resources for Young Investigators.” BMC Rheumatology 7, no. 1: 2. 36895053 10.1186/s41927-023-00327-w PMC9996937

[jlcd70096-bib-0003] Atchley, R. A. , L. Halderman , K. Kwasny , and L. Buchanan . 2003. “The Processing of Pseudohomophones by Adults With a History of Developmental Language Disabilities.” Brain and Cognition 53, no. 2: 139–144. 14607134 10.1016/s0278-2626(03)00096-4

[jlcd70096-bib-0004] Bauer, M. S. , L. Damschroder , H. Hagedorn , J. Smith , and A. M. Kilbourne . 2015. “An Introduction to Implementation Science for the Non‐Specialist.” BMC Psychology 3: 1–12. 26376626 10.1186/s40359-015-0089-9 PMC4573926

[jlcd70096-bib-0005] Bishop, D. V. 2010. “Which Neurodevelopmental Disorders Get Researched and Why?” PLoS ONE 5, no. 11: e15112. 21152085 10.1371/journal.pone.0015112 PMC2994844

[jlcd70096-bib-0006] Bishop, D. V. , M. J. Snowling , P. A. Thompson , et al. 2017. “Phase 2 of CATALISE: A Multinational and Multidisciplinary Delphi Consensus Study of Problems With Language Development: Terminology.” Journal of Child Psychology and Psychiatry 58, no. 10: 1068–1080. 28369935 10.1111/jcpp.12721 PMC5638113

[jlcd70096-bib-0007] Botting, N. 2020. “Language, Literacy and Cognitive Skills of Young Adults With Developmental Language Disorder (DLD).” International Journal of Language & Communication Disorders 55, no. 2: 255–265. 31994284 10.1111/1460-6984.12518

[jlcd70096-bib-0008] Chalmers, I. , and P. Glasziou . 2009. “Avoidable Waste in the Production and Reporting of Research Evidence.” Lancet 374, no. 9683: 86–89. 19525005 10.1016/S0140-6736(09)60329-9

[jlcd70096-bib-0009] Dunham, R. 2006. "Nominal Group Technique: A User's Guide." University of Wisconsin.

[jlcd70096-bib-0010] Edgell, C. , and A. Rosenberg . 2022. “Putting Plain Language Summaries Into Perspective.” Current Medical Research and Opinion 38, no. 6: 871–874. 35400253 10.1080/03007995.2022.2058812

[jlcd70096-bib-0011] Elo, S. , and H. Kyngäs . 2008. “The Qualitative Content Analysis Process.” Journal of Advanced Nursing 62, no. 1: 107–115. 10.1111/j.1365-2648.2007.04569.x. 18352969

[jlcd70096-bib-0012] Fisher, R. J. , F. Riley‐Bennett , L. Russell , et al. 2021. “Nominal Group Technique to Establish the Core Components of Home‐Based Rehabilitation for Survivors of Stroke With Severe Disability.” BMJ Open 11, no. 12: e052593. 10.1136/bmjopen-2021-052593 PMC8640659 34857570

[jlcd70096-bib-0013] Gainey, K. M. , J. Smith , K. J. McCaffery , S. Clifford , and D. M. Muscat . 2023. “What Author Instructions do Health Journals Provide for Writing Plain Language Summaries? A Scoping Review.” Patient‐Patient‐Centered Outcomes Research 16, no. 1: 31–42. 36301440 10.1007/s40271-022-00606-7 PMC9813023

[jlcd70096-bib-0014] Gasparini, L. , S. Ziegenfusz , N. Turner , S. Leitão , M. C. St Clair , and E. Jackson . 2025. “How to Create Accessible Research Summaries for the Developmental Language Disorder Community.” International Journal of Language & Communication Disorders 60, no. 1: e13142. 39625399 10.1111/1460-6984.13142

[jlcd70096-bib-0015] Greenhalgh, T. M. , J. Bidewell , E. Crisp , J. Warland , and G. Dermody . 2023. Understanding Research Methods for Evidence‐Based Practice in Health. John Wiley & Sons.

[jlcd70096-bib-0016] Harold, M. 2019. “The Research Translation Problem: A Modest Proposal.” ASHA Leader 24, no. 7: 52–61. 10.1044/leader.FTR2.24072019.52.

[jlcd70096-bib-0017] Hersh, L. , B. Salzman , and D. Snyderman . 2015. “Health Literacy in Primary Care Practice.” American Family Physician 92, no. 2: 118–124. 26176370

[jlcd70096-bib-0018] Hinckley, J. , and C. El‐Khouri . 2023. “Why and How to Publish Aphasia‐Friendly Research Summaries.” Journal of Communication Disorders 104: 106338. 37192574 10.1016/j.jcomdis.2023.106338

[jlcd70096-bib-0019] Ibrahim, A. M. 2018. “ Use of a Visual Abstract to Disseminate Scientific Research .” Available at: https://www.cirtc.org/tools-resourcesdatabase/visual-abstract-dissemination. 10.1038/ajg.2017.268 28925990

[jlcd70096-bib-0020] King, E. E. , E. C. Borman‐Shoap , W. Sveen , A. D. Wolfe , and M. B. Pitt . 2023. “Creating Video Abstracts for Scholarly Dissemination.” Academic Medicine 98, no. 1: 151. 35731590 10.1097/ACM.0000000000004784

[jlcd70096-bib-0021] Koontz, A. , J. Duvall , R. Johnson , T. Reissman , and E. Smith . 2022. “‘Nothing About Us Without Us:’ Engaging at Users in at Research.” Assistive Technology 34, no. 5: 499–500. 36170692 10.1080/10400435.2022.2117524

[jlcd70096-bib-0022] Lundberg, D. J. , and J. A. Chen . 2024. “Structural Ableism in Public Health and Healthcare: A Definition and Conceptual Framework.” Lancet Regional Health–Americas 30: 100650. 38188095 10.1016/j.lana.2023.100650 PMC10770745

[jlcd70096-bib-0023] Marsden, E. , I. Alferink , S. Andringa , et al. 2018. “ Open Accessible Summaries in Language Studies (OASIS) [Database] .” Available at: https://www.oasis‐database.org.

[jlcd70096-bib-0024] McGregor, K. K. 2020. “How We Fail Children With Developmental Language Disorder.” Language, Speech, and Hearing Services in Schools 51, no. 4: 981–992. 10.1044/2020_LSHSS-20-00003. 32755505 PMC7842848

[jlcd70096-bib-0025] Montgomery, J. W. , R. B. Gillam , and J. L. Evans . 2021. “A New Memory Perspective on the Sentence Comprehension Deficits of School‐Age Children With Developmental Language Disorder: Implications for Theory, Assessment, and Intervention.” Language, Speech, and Hearing Services in Schools 52, no. 2: 449–466. 33826402 10.1044/2021_LSHSS-20-00128 PMC8711711

[jlcd70096-bib-0026] Mountford, H. S. , R. Braden , D. F. Newbury , and A. T. Morgan . 2022. “The Genetic and Molecular Basis of Developmental Language Disorder: A Review.” Children 9, no. 5: 586. 35626763 10.3390/children9050586 PMC9139417

[jlcd70096-bib-0027] Norbury, C. F. , D. Gooch , C. Wray , et al. 2016. “The Impact of Nonverbal Ability on Prevalence and Clinical Presentation of Language Disorder: Evidence From a Population Study.” Journal of Child Psychology and Psychiatry 57, no. 11: 1247–1257. 27184709 10.1111/jcpp.12573 PMC5082564

[jlcd70096-bib-0028] Palinkas, L. A. , S. M. Horwitz , C. A. Green , J. P. Wisdom , N. Duan , and K. Hoagwood . 2015. “Purposeful Sampling for Qualitative Data Collection and Analysis in Mixed Method Implementation Research.” Administration and Policy in Mental Health and Mental Health Services Research 42: 533–544. 24193818 10.1007/s10488-013-0528-y PMC4012002

[jlcd70096-bib-0029] Qualtrics . 2024, June. *Qualtrics Survey Software* [Computer software]. Qualtrics. https://www.qualtrics.com.

[jlcd70096-bib-0030] Rose, D. , and A. Meyer . 2002. “Teaching Every Student in the Digital Age: Universal Design for Learning.” Association for Supervision and Curriculum Development.

[jlcd70096-bib-0031] Rosenberg, A. , J. Walker , S. Griffiths , and R. Jenkins . 2023. “Plain Language Summaries: Enabling Increased Diversity, Equity, Inclusion and Accessibility in Scholarly Publishing.” Learned Publishing 36, no. 1: 109–118.

[jlcd70096-bib-0033] Snowling, M. J. , F. J. Duff , H. M. Nash , and C. Hulme . 2016. “Language Profiles and Literacy Outcomes of Children With Resolving, Emerging, or Persisting Language Impairments.” Journal of Child Psychology and Psychiatry 57, no. 12: 1360–1369. 26681150 10.1111/jcpp.12497 PMC5132029

[jlcd70096-bib-0034] St Clair, M. C. , J. Horsham , V. Lloyd‐Esenkaya , et al. 2023. “The Engage With Developmental Language Disorder (E‐DLD) Project: Cohort Profile.” International Journal of Language & Communication Disorders 58, no. 3: 929–943. 36565246 10.1111/1460-6984.12835

[jlcd70096-bib-0035] Stoll, M. , M. Kerwer , K. Lieb , and A. Chasiotis . 2022. “Plain Language Summaries: A Systematic Review of Theory, Guidelines and Empirical Research.” PLoS ONE 17, no. 6: e0268789. 35666746 10.1371/journal.pone.0268789 PMC9170105

